# The nedd-8 activating enzyme gene underlies genetic resistance to infectious pancreatic necrosis virus in Atlantic salmon

**DOI:** 10.1016/j.ygeno.2021.09.012

**Published:** 2021-11

**Authors:** Jon Pavelin, Ye Hwa Jin, Remi L. Gratacap, John B. Taggart, Alastair Hamilton, David W. Verner-Jeffreys, Richard K. Paley, Carl-johan Rubin, Stephen C. Bishop, James E. Bron, Diego Robledo, Ross D. Houston

**Affiliations:** a The Roslin Institute and Royal (Dick) School of Veterinary Studies, University of Edinburgh, Midlothian EH25 9RG, UK; b Institute of Aquaculture, School of Natural Sciences, University of Stirling, FK9 4LA, UK; c Hendrix Genetics RTC, Villa ’de Körver’, Spoorstraat, 695831 CK Boxmeer, the Netherlands; d Centre for Environment, Fisheries and Aquaculture Science (Cefas), Weymouth Laboratory, Dorset DT4 8UB, UK; e Department of Medical Biochemistry and Microbiology, Uppsala University, Sweden

**Keywords:** QTL, Disease resistance, CRISPR, Whole genome resequencing, Gene expression, Aquaculture

## Abstract

Genetic resistance to infectious pancreatic necrosis virus (IPNV) in Atlantic salmon is a rare example of a trait where a single locus (QTL) explains almost all of the genetic variation. Genetic marker tests based on this QTL on salmon chromosome 26 have been widely applied in selective breeding to markedly reduce the incidence of the disease. In the current study, whole genome sequencing and functional annotation approaches were applied to characterise genes and variants in the QTL region. This was complemented by an analysis of differential expression between salmon fry of homozygous resistant and homozygous susceptible genotypes challenged with IPNV. These analyses pointed to the NEDD-8 activating enzyme 1 (*nae1*) gene as a putative functional candidate underlying the QTL effect. The role of *nae*1 in IPN resistance was further assessed via CRISPR-Cas9 knockout of the *nae*1 gene and chemical inhibition of the nae1 protein activity in Atlantic salmon cell lines, both of which resulted in highly significant reduction in productive IPNV replication. In contrast, CRISPR-Cas9 knockout of a candidate gene previously purported to be a cellular receptor for the virus (*cdh*1) did not have a major impact on productive IPNV replication. These results suggest that *nae*1 is the causative gene underlying the major QTL affecting resistance to IPNV in salmon, provide further evidence for the critical role of neddylation in host-pathogen interactions, and highlight the value in combining high-throughput genomics approaches with targeted genome editing to understand the genetic basis of disease resistance.

## Highlights

• Whole genome sequencing and functional annotation were used to characterise the major IPNV resistance QTL in Atlantic salmon.
• Gene expression analyses pointed to NEDD-8 activating enzyme 1 (nae1) as a putative functional candidate underlying the QTL.
• CRISPR-Cas9 knockout and chemical inhibition of nae1 in cell lines resulted in significant reduction in IPNV replication.
• CRISPR-Cas9 knockout of a previously suggested candidate gene (cdh1) did not have an impact on productive IPNV replication.
• These results suggest that nae1 is the causative gene underlying the major QTL affecting resistance to IPNV in salmon.

## 1 Background

Understanding the genetic regulation of traits of importance to farmed animal production is key to guiding optimal use of genomic information in selective breeding programmes [1,2]. Such production traits are typically underpinned by a polygenic architecture, with many loci of minor effect contributing to their heritability [1,2]. However, there are exceptions where major effect loci segregate within farmed animal populations, and a single genomic region underlies the majority of genetic variation in a trait of interest. One such example is the case of host resistance to infectious pancreatic necrosis virus (IPNV) in Atlantic salmon, a species with a global aquaculture production of >2.4 million tonnes, worth >$17.1 billion USD in 2018 [3]. A major quantitative trait locus (QTL) affecting resistance was described by two independent groups [4,5], and explains 80–100% of the genetic variance in mortality due to the disease. The application of marker-assisted selection for the identified resistance allele has exemplified the benefits to be gained from applied molecular genetics, contributing to a reduction in IPN mortalities from tens-of-millions in 2009 down to negligible levels five years later [6]. However, while the epithelial cadherin gene (*cdh1*) has been previously suggested to play a role in mediating the QTL effect [7], there are still significant knowledge gaps in the underlying functional mechanisms underlying the QTL. Identification of functional mechanisms and variants leads to new opportunities for disease control [[8], [9], [10], [11]], including genome editing to introduce resistance to salmonid strains or species which do not carry the major resistance allele for the QTL.

IPNV is the prototypical birnavirus (genus *Aquabirnaviridae*, family *Birnaviridae*), and consists of an unenveloped capsid containing a bisegmented double-strand RNA genome. IPNV is capable of causing high levels of morbidity and mortality in farmed salmonid species, including Atlantic salmon (*Salmo salar*) and rainbow trout (*Oncorhynchus mykiss)* [12]. Clinical signs of IPNV include pancreatic necrosis accompanied by abdominal swelling, darkening of the skin and erratic swimming behaviour. IPNV outbreaks typically occur at two distinct points of the salmon aquaculture production cycle; in first feeding fry in freshwater and in smolts after transfer to seawater [12]. Protection during freshwater production can be partially achieved through vigilant monitoring and biosecurity, but this is ineffective in open seawater pens due to constant exposure to the ocean environment. Vaccination is also partially effective, but generally only feasible for helping prevent disease in the later lifecycle post-smolt stage of production [13].

A large and significant genetic component to IPN resistance at both crucial stages of the salmon lifecycle has been demonstrated [[14], [15], [16]], and the major QTL explaining most of this genetic variation has been well described in both Scottish [4,[17], [18], [19]] and Norwegian strains [5,7], with evidence for at least partial dominance of the resistance-associated allele [5,17]. The *cdh1* gene was suggested to play a functional role in host resistance to IPNV via prevention of entry of the virus into cells [7]. However, the purported functional mutation in this gene was only in partial linkage disequilibrium with the inferred QTL genotype (r^2^ ~ 0.58) meaning that significant other factors must contribute to the QTL effect. Furthermore, the proposed mechanism of an amino acid change in Cdh1 preventing viral entry to the cells seems unlikely since IPNV can successfully replicate in fully resistant salmon fry [20]. Furthermore, the mechanism of viral entry into cells has now been demonstrated to be micropinocytosis [21], which is inconsistent with the proposed clathrin-mediated endocytosis associated with *cdh1* [7]. CRISPR-Cas9 genome editing provides new opportunities to assess the function of candidate genes underlying this QTL, and may also lead to avenues for application to aquaculture via transfer of the IPN resistance mechanism across salmonid species [9], including to rainbow trout.

In this study, pooled whole genome sequencing of RR (homozygous resistant) and SS (homozygous susceptible) salmon fry was used to discover and functionally annotate all polymorphisms within the QTL region. Host transcriptomic and viral load analysis were performed on RR and SS fry from two families, based on whole fry samples collected at selected timepoints pre- and post-IPNV challenge. Fine mapping using the pooled whole genome sequencing highlighted a number of highly significant SNPs in the QTL region, which clustered around the coding region and putative regulatory regions of the gene NEDD-8 activating enzyme 1 (*nae1*). Furthermore, *nae1* was one of the most significantly differentially expressed genes between RR and SS fry genome-wide, showing notably higher expression levels in resistant fish both constitutively and post-challenge. Following these findings, a series of experiments to disrupt the activity of *nae1* and *cdh1* were performed in Atlantic salmon cell lines, using CRISPR-Cas9 knockout and specific molecular inhibitors. The results point to a major role for *nae1* but not *cdh1* in viral replication in salmon cells, lending significant support to the hypothesis that *nae1* is a functional gene mediating the large effect of the QTL on resistance to the virus.

## 2 Results

### 2.1 Fine mapping of IPN resistance QTL using whole genome sequence data

**Fig. 1:**
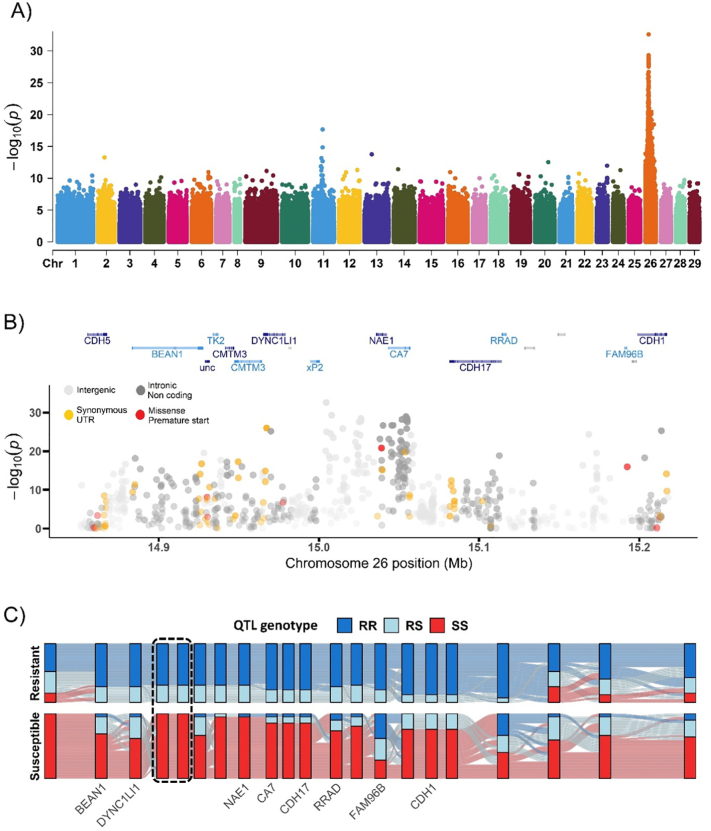
Genetic mapping and functional characterisation of the IPN resistance QTL region; A) Manhattan plot showing association between genome-wide SNPs and QTL genotype, B) map of annotated genes and functional annotation of SNPs within the most significant QTL region; C) The concordance between significant SNP genotypes and inferred QTL genotypes in offspring from double heterozygous parent families. Each vertical bar represents a SNP in or around the QTL region and each horizontal line represents an individual animal. The boxed area comprises two of the most significant SNPs from the genome-wide scan, and the SNPs that show full concordance between QTL genotype and SNP genotype in susceptible homozygous animals. There are no SNPs with full concordance between QTL genotype and SNP genotype in resistant homozygous animals.

To fine map the IPN resistance QTL, and to identify candidate functional genes and polymorphisms, genomic DNA from salmon fry of known QTL genotype was pooled and whole genome sequencing was performed. These fry were selected from two large IPNV challenge experiments performed on salmon fry in 2007 and 2008. Families where both parents were heterozygous for the QTL were identified (*n* = 11 in 2007, and *n* = 12 in 2008), and from each of those families two homozygous resistant (RR) fish and two homozygous susceptible (SS) fish (total *n* = 22 in 2007, and total *n* = 24 in 2008) were selected for pooling of genomic DNA at equimolar concentrations and sequencing (2 x pools of RR fish and 2 x pools of SS fish, sequence reads available at NCBI Short Read Archive PRJNA614520) Following alignment of sequence reads to the Atlantic salmon reference genome (GenBank accession GCA_000233375.4), variants were called and the allele frequency differences between the RR and SS pools were calculated (Fig. 1A). The QTL region of chromosome 26 contained the vast majority of the most significant SNPs, with a notable peak at approximately 15 Mb in an intergenic region upstream of the nedd-8 activating enzyme E1 (*nae1*) gene (Fig. 1A, B).

To screen for putative functional candidate SNPs and indels within the region of the QTL the predicted consequence of all variants was assessed using the SNPEFF software [22]. Two missense mutations were identified within the QTL region, one in the epithelial cadherin locus (*cdh1*) previously identified by Moen et al. [7]*,* and one in the *nae1* locus, which has not previously been reported (Fig. 1B).

To further assess the association between selected high priority SNPs dispersed throughout the QTL region and the putative QTL genotype, a KASP assay was developed for 21 polymorphisms which were subsequently genotyped in individual samples of RR and SS genotypes used in the pooled sequencing experiment. There was no single SNP or indel that showed a perfect concordance with the putative underlying QTL genotype, which is in agreement with Moen et al [7]. However, there were two SNPs in the intergenic region at ~15 Mb which showed a pattern where all genotyped SS fish across two yeargroups of the breeding population were homozygous for one allele, RR fish were either homozygous for the alternative allele or heterozygous (Fig. 1C, Supplementary File 1), and heterozygous parents were either heterozygous at the SNPs or fixed for the susceptibility-associated SNP allele (data not shown). This pattern is consistent with a dominant-acting primary locus at this location, where a single copy of the resistance-associated allele was sufficient to ensure fish were fully resistant (i.e. survived challenge with IPNV), but also suggests the possibility of a secondary locus acting in the QTL region.

### 2.2 Contrast in nae1 gene expression between resistant and susceptible salmon fry

In order to shortlist candidate genes in the QTL region that may be causative for IPN resistance, global gene expression analyses were performed in RR and SS genotyped individuals from families where both parents were heterozygous for the QTL (families B and C in Houston et al [17]). To achieve this, replicate family-specific tanks (*n* = 50 per tank) were immersion-challenged with IPNV as described in Robledo et al [20], and whole fry were sampled pre-challenge, 24 h post-challenge, and 7 days post-challenge. Fry were assigned their QTL genotype using the microsatellite marker panel described in Houston et al [17], and RR and SS homozygous fry were chosen for gene expression analyses. Whole fry were homogenised, pooled in quadruplicate, and total RNA was extracted.

**Fig. 2:**
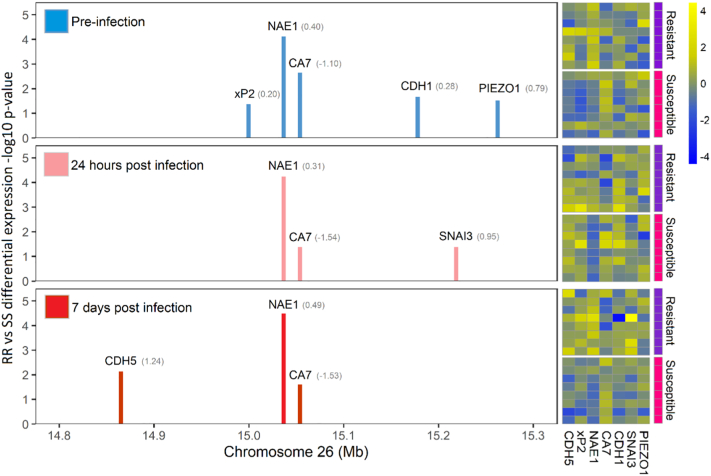
Differential expression of genes in the IPN resistance QTL region in salmon fry pre-challenge, 1 day post challenge, and 7 days post challenge. The nae1 gene is consistently the most significant differentially expressed gene in the QTL region at all timepoints. The values in parentheses represent the fold change values for the genes. The information on the QTL region genes is taken from the following microarray probes: CDH5: Ssa#DW552050; xP2: Ssa#S32001422; NAE1: Ssa#STIR01613; CA7: Ssa#S35540993; CDH1: Ssa#S35660729; SNAI3: Omy#BX299558; PIEZO1: Ssa#DY703210 (Supplementary File 2). The heatmap on the right shows the relative expression levels of these probes in individual samples, contrasting RR (Resistant) and SS (Susceptible) fry. Details of the samples used in the microarray experiment are given in Supplementary File 3, while the full raw data are given in Supplementary File 4. Differential expression of genes in the IPN resistance QTL region in salmon fry pre-challenge, 1 day post challenge, and 7 days post challenge. The nae1 gene is consistently the most significant differentially expressed gene in the QTL region at all timepoints. The values in parentheses represent the fold change values for the genes. The information on the QTL region genes is taken from the following microarray probes: CDH5: Ssa#DW552050; xP2: Ssa#S32001422; NAE1: Ssa#STIR01613; CA7: Ssa#S35540993; CDH1: Ssa#S35660729; SNAI3: Omy#BX299558; PIEZO1: Ssa#DY703210 (Supplementary File 2). The heatmap on the right shows the relative expression levels of these probes in individual samples, contrasting RR (Resistant) and SS (Susceptible) fry. Details of the samples used in the microarray experiment are given in Supplementary File 3, while the full raw data are given in Supplementary File 4.

Global gene expression analyses of pooled ‘RR’ and ‘SS’ individuals revealed that *nae1* was the most significant differentially expressed gene within the QTL region (Fig. 2), and one of the most significant genome wide during IPNV infection (Supplementary File 2). Interestingly, *nae1* expression was consistently higher in QTL-resistant fry than in QTL-susceptible fry at all timepoints, including constitutively higher expression pre-challenge (Fig. 2).

### 2.3 IPN virus replicates in both resistant and susceptible fish

Viral load in RR, RS and SS IPNV-challenged fry from families B and C was assessed at day 1, day 7, and day 21 post challenge. Viral load was found to be between 1 and 2 log lower in RR and RS individuals compared with SS individuals, but all genotypes have viral load that indicate productive replication of the virus (Supplementary Fig. 1). This is consistent with previous reports by Reyes-Lopez et al [23] and Robledo *et al* [20] in both head kidney and whole fry, which showed an appreciable increase in viral load in fry from both fully resistant and susceptible families during an IPNV challenge. These data demonstrate that the mechanism underlying genetic resistance is not prevention of entry of the virus to the cell, nor the complete prevention of viral replication within the cell.

### 2.4 CRISPR knockout of nae1 markedly reduces IPNV replication in salmon cells

Nae1 is an enzyme that is responsible for covalently linking ubiquitin-like protein Nedd8 to target proteins, often modifying their function [24]. Inhibition of nae1 activity using a small molecular inhibitor (MLN4924) has been shown to have broad-acting anti-viral activity and to inhibit the replication of several DNA and RNA viruses in vitro, highlighting the importance of the neddylation process during viral infection [25]. To assess the role of nae1 in IPNV replication in Atlantic salmon cells, two complementary approaches were taken using the Salmon head kidney (SHK-1) cell line; CRISPR-Cas9 knockout (KO) of the *nae*1 gene, and MLN4924 inhibition of the nae1 protein activity.

**Fig. 3:**
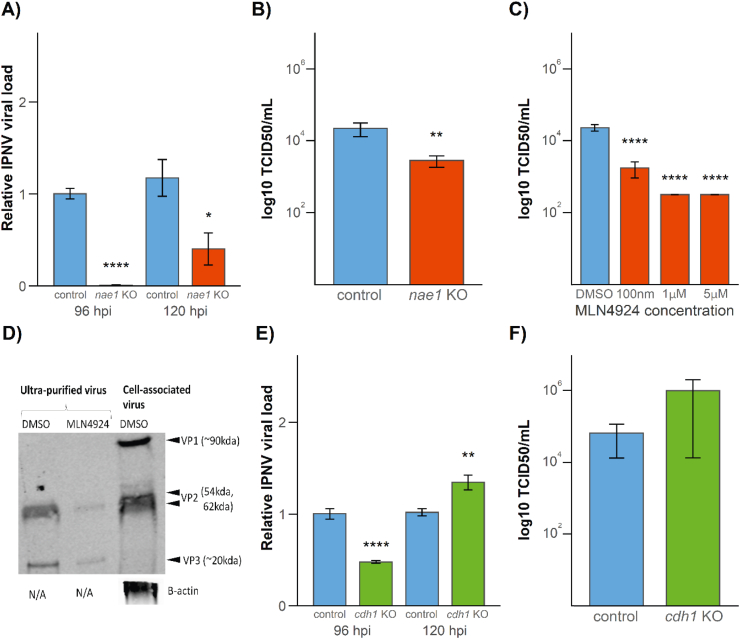
Assessment of the role of Nae1 and Cdh1 in the replication of IPNV in Atlantic salmon cells. A) IPNV viral load at 96 and 120 hpi in control and nae1 KO SHK-1 infected with IPNV at an MOI of 0.01. Relative expression levels of IPNV VP2 to ef1a in cells were normalised to time-matched control SHK-1 cells. B) Infectivity of IPNV in supernatant at 120 hpi in control and nae1 KO SHK-1 infected with IPNV at an MOI of 0.01 was assessed by TCID50/mL on naïve CHSE-214 cells. C) Infectivity of IPNV in cells and supernatant at 120 hpi in SHK-1 cells treated with 100 nM, 1 μM and 5 μM of MLN4924 or DMSO only and infected with IPNV at an MOI of 0.01 was assessed by TCID50/mL on naïve CHSE-214 cells. D) IPNV viral protein in supernatant of SHK-1 cells treated with 100 nM MLN4924 and infected at an MOI of 0.01 at 120 hpi was analysed by western blotting using an antibody against IPNV viral proteins. E) IPNV viral load at 120 hpi in control and cdh1 KO SHK-1 infected with IPNV at an MOI of 0.01. Relative expression levels of IPNV VP2 to ef1a in cells were normalised to time-matched control SHK-1 cells. F) Infectivity of IPNV in supernatant at 120 hpi in control and cdh1 KO SHK-1 infected with IPNV at an MOI of 0.01 was assessed by TCID50/mL on naïve CHSE-214 cells. Significance levels denoted: * *P* < 0.05, ** *P* < 0.01, **** *P* < 0.0001.

Firstly, CRISPR-Cas9 genome editing was used to KO the *nae1* gene in SHK-1 cells using recombinant Cas9 protein and custom synthesised gRNAs; a method for high specificity editing of target genes in salmonid cell cultures [26]. Exon 2 of the Atlantic salmon *nae1* locus was targeted and editing efficiency was 93–97% resulting in 82–87% frameshift mutation (depending on the replicate), highlighting that the vast majority of cells in the mixed cell population were successfully edited. Nonetheless, it should be noted that herein the use of ‘KO’ refers to a mixed population of cells where the majority of cells carry a KO-causing edit, while a minority of cells will still produce functional protein. Following IPNV challenge at a multiplicity of infection (MOI) of 0.01, IPNV RNA load and productive viral output were assessed by qRT-PCR and TCID50 assays, respectively. The viral load in the *nae1* KO SHK-1 cell cultures at 96 and 120 hpi was 109.6 and 2.7-fold lower (respectively) than mock-challenged control SHK-1 cells (Fig. 3A, p < 0.001 and 0.05). In addition, the infectivity of viral output in the supernatants at 120 hpi was 7.8-fold lower in *nae1* KO cells (Fig. 3B, p < 0.01).

Secondly, the MLN4924 small molecule inhibitor of nae1 was used in the Atlantic salmon SHK-1 cell line to inhibit nae1 protein function. Cells were treated with 100 nM MLN4924 dissolved in DMSO, or DMSO only as a negative control, for 24 h prior to infection with IPNV and measurements of viral load and output were taken as described above. Despite little difference in IPNV RNA copy number during the course of infection (Supplementary Fig. 2), there was a substantial (13 to 73-fold) decrease in viral output as measured at 120 hpi in SHK-1 cells (Fig. 3C). In order to confirm this decrease in viral output, western blot against viral proteins was performed on virus purified from SHK-1 cells treated with MLN4924 or DMSO at 120 h (Fig. 3D). There was a notable decrease in the abundance of IPNV viral proteins in cells treated with MLN4924, which demonstrated that inhibition of nae1 activity results in a decrease in viral output. There was no associated decrease in cell viability with the MLN4924 treatment compared to DMSO-treated controls (Supplementary Fig. 3).

### 2.5 Cdh1 is not required for IPNV infection and replication in salmon cells

The IPN resistance QTL was independently reported by Moen et al [5] and subsequently the resistance phenotype was partially attributed to a missense variant in the *cdh1–1* gene, which encodes a cell surface receptor [7]. This gene was posited to encode a protein which is required for entry of IPNV into cells. To test this hypothesis and assess the putative role of cdh1 in IPNV infection, *cdh1* KO SHK-1 cells were generated using CRISPR-Cas9 genome editing using the method described above [27]. Using a guide RNA that targets exon 2 of *cdh1–1*, an editing efficiency of 90–94% was observed, resulting in 90–93% frameshift mutation rate (depending on the replicate) in the SHK-1 cells. If cdh1 was critical for the entry and replication of IPNV, viral entry is likely to be prevented in knockout cells, and a marked reduction in viral load in the edited cell culture would be expected. However, while there was a minor (2.1-fold) decrease in viral load measured by qPCR compared to controls at 96 hpi, there was a small increase (1.3-fold) compared to controls at 120 hpi (Fig. 3E). Furthermore, there was no difference in productive viral output between *cdh1* KO and control cells as analysed by TCID50 assays (Fig. 3F). This indicates that cdh1 is not essential for the entry of IPNV into salmon cells, nor for successful IPNV replication and productive viral output in these cell lines. To further assess the role of cdh1 in IPN resistance, specific antibodies against the extracellular domain of cdh1 (as used in Moen et al [7]) were used to assess whether they block IPNV infection and replication in salmon cells. Despite effective and striking neutralisation of IPNV infection with a specific antibody against IPNV viral protein VP2, there was no indication of an impact of the anti-cdh1 antibody on IPNV replication in the SHK-1 cells (Supplementary Fig 4).

## 3 Discussion

It has been well documented that resistance to IPN in Atlantic salmon has a major genetic component, and the majority of variation in mortality observed between resistant and susceptible fish can be explained by a major QTL on chromosome 26 [4,5]. However, the causative mutations and the underlying molecular biology of the resistance phenotype were not well understood. In the current study, whole genome sequencing of salmon fry with known QTL genotypes was used to fine map the most significant SNPs and indels to a region upstream of the *nae1* gene. Global gene expression profiling highlighted differentially expressed between susceptible and resistant fish prior to and during IPNV infection, and this revealed that *nae1* is one of the most significant differentially expressed genes genome-wide, and the most significant in the QTL region (Supplementary File 2). Finally, the perturbation of the two primary candidate genes within the IPN QTL was tested using salmon cell line models; *cdh1* (as proposed by Moen et al. [7]), and *nae1* based on evidence presented in the current study.

The whole genome resequencing revealed two missense coding mutations, one in *nae1* and one in *cdh1*. The genotyping results highlighted that no single SNP or indel was fully concordant with the QTL genotype, but a cluster of SNPs in this region were all found to be homozygous for one allele in susceptible fish, and either heterozygous or homozygous for the alternative allele in resistant fish. These findings may be consistent with local epistasis, with a dominant acting primary resistance locus, or with a further (unidentified) secondary locus or loci in the region associated with the QTL effect in fish fixed for the susceptibility allele at this primary locus. These findings are generally consistent with results using a similar approach by Moen et al., although the location of the most significant SNPs differs [7], and highlight that no single SNP marker can be used to accurately assign QTL status across populations. The results of the gene expression comparison showed *nae1* to be one of the most significant differentially expressed genes between susceptible and resistant fish, both in the pre-challenge fry and at all measured timepoints post challenge. This highlights the possibility that the intergenic region located ~15 Mb on chromosome 26 contains regulatory elements for *nae1* expression, or that the *nae1* missense SNP alters the expression of the gene (either directly or indirectly). Interestingly although higher expression was associated with resistance in these IPNV challenged fry, the downstream functional experiments suggest that lack of functional nae1 activity is linked to reduction in productive viral replication. Further investigation into the molecular mechanisms underlying the functional impact of *nae1* perturbation is required to understand this phenomenon further. In addition, other genes within the QTL region may form a component of genetic resistance, such as *ca7* which shows consistent gene expression differences between RR and SS genotypes in the opposite direction to *nae1*, and could be further investigated using genome editing and functional virology experiments.

Nae1 is an enzyme responsible for the covalent attachment of nedd8, an ubiquitin like modifier, to substrate proteins. Neddylation primarily functions to activate the cullin-RING ligases that in turn regulate the degradation of specific substrates via ubiquitination [24]. Using a small molecular inhibitor of nae1, neddylation has been shown to be important in the context of host-pathogen interaction for several DNA and RNA viruses [25]. Le-Trilling et al found that nae1 inhibition reduced the replication of human cytomegalovirus, mouse cytomegalovirus, herpes simplex virus 1, and influenza B virus [25]. In the current study, a similar effect was observed for IPNV in Atlantic salmon, with nae1 knockout or chemical inhibition resulting in significant decrease in productive viral replication (Fig. 3). Neddylation plays a significant role in the stimulation of the host type 1 interferon response to viral infections, and many viruses attempt to evade the host immune response by targeting type I interferon signalling [28]. The mechanisms by which IPNV evades immune response include actions of the viral proteins to interfere with IRF3, IRF7, and NF-kB signalling [[29], [30], [31]], all of which act to stimulate type 1 interferon signalling. Interestingly, neddylation has been shown to be a critical component of the antiviral response in zebrafish, and was postulated to act via type 1 interferon response activation by IRF3 and IRF7 [32]. Interestingly, both IRF3 and IRF7 were amongst the most significantly differentially expressed genes between RR and SS fry following IPNV challenge in the current study, showing higher expression in susceptible fish, and highlighting their importance in IPNV host response (Supplementary File 2). It is conceivable that the genetic variants identified in the *nae*1 regulatory or coding regions lead to an alteration of neddylation function in resistant fish. As a consequence this would result in a modified type 1 interferon response, potentially due to changes in IRF3 and IRF7 signalling, which may protect IPNV infected fish from the damaging cytokine storm which is postulated to be a major cause of IPN morbidity [33].

A SNP within the e-cadherin gene (*cdh1*) has previously been proposed as a functional variant which leads to IPN resistance in Atlantic salmon [7]. The proposed mechanism was that Cdh1 acts as the receptor for IPNV to enter cells via clathrin-mediated endocytosis, and the causative SNP blocks IPNV binding and entry. While IPNV has been shown to bind to cdh1 [7], it is unlikely that this is the sole route of entry during infection. Reyes-Lopez et al. [23], Robledo et al. [20], and the findings presented herein (Supplementary Fig. 1) show that resistant fish do become infected with IPNV and with viral load levels that can only be explained by successful replication in cells of fully (homozygous) resistant salmon fry. It has also recently been demonstrated that macropinocytosis is the primary route for IPNV entry into SHK-1 and CHSE-214 cells, a process that is likely to be non-discriminatory and not reliant on a specific receptor [21,34]. To assess this further in the current study, CRISPR-Cas9 editing was used to knockout *cdh1* with high efficiency in salmon cell culture. When these KO cells were challenged with IPNV there was no consistent impact on viral load, and no impact on productive viral output when compared with wild-type cells, indicating that cdh1 is not essential for viral entry or replication in these cells (Fig. 3E). Furthermore, using an antibody against e-cadherin, it was not possible to block IPNV entry or inhibit replication in Atlantic salmon cells in culture in the current study (Supplementary Fig 4). While it is plausible that cdh1 plays a role in IPN resistance at the site of infection in vivo (e.g. in gut epithelia), the results presented herein do not support a major role for cdh1 in IPN resistance. In contrast, the genetic mapping, gene expression, and functional virology experiments all provide evidence for a major role of nae1 in underlying the major IPN resistance QTL.

The IPN QTL has become a well-known exemplar of the application of molecular genetics to tackle a major infectious disease problem in a farmed animal [1,6]. Application of marker-assisted selection for the resistance allele has reduced incidence of disease outbreaks close to zero in all the major salmon-producing countries [1,6]. While identification of the underlying causative gene and mechanisms is of limited practical utility to disease control in salmon aquaculture, IPN is also a serious pathogen of other salmonid species, including rainbow trout. Unlike salmon, there is no evidence for an equivalent major QTL affecting IPN resistance segregating in commercial rainbow trout populations, albeit the trait is heritable and QTL of smaller effect have been identified [35,36]. However, the identification of the putative causative resistance gene in salmon, combined with the advances in genome editing technology in aquaculture [9], gives rise to new opportunities for cross-species transfer of resistance mechanisms. For example, modulation of gene expression of *nae1* using dead Cas9 systems may affect IPNV replication and resistance in trout cells, or introgression-by-editing [9] of DNA sequence templates corresponding to salmon resistance alleles into trout is a worthy future avenue to explore.

## 4 Conclusions

Fine mapping of the major IPNV resistance QTL using whole genome sequencing combined with differential expression between homozygous resistant and homozygous susceptible fish both pointed to *nae1* as a strong candidate causative gene. Functional assessment of CRISPR-Cas9 knockout of *nae1*, and specific inhibition of the nae1 protein activity in IPNV-challenged salmon cells revealed a marked decrease in productive viral output. A previously identified candidate gene *cdh1* has been suggested to be the cellular receptor for IPNV, with resistance due to prevention of viral entry to cells. However, in the current study, prevention of IPNV binding to cdh1 either via CRISPR-Cas9 knockout of *cdh1* or binding of a cdh1 antibody did not influence productive IPNV replication. Taken in combination, these results show that *nae1* is the likely causative gene underlying the major IPN QTL, which further highlights the extensive role of neddylation in immune response to a broad range of viral infections. The study also highlights the value of combining high-throughput genomics approaches with targeted genome editing to identify functional genes underlying an important aquaculture production trait.

## 5 Materials and methods

### 5.1 DNA sequencing and fine mapping

23 nuclear families from two yeargroups, derived from a commercial salmon breeding programme (Landcatch strain, Hendrix Genetics) and where both sire and dam were heterozygous for the IPN resistance QTL, were identified using the methods described in Houston et al. [17]. The Landcatch strain was derived from a series of historical crosses between Scottish wild salmon and Norwegian breeding programme strains. From each of these families, two fry homozygous for the resistant allele (RR) and two fry homozygous for the susceptibility allele (SS) were identified. Four groups were then established, RR fry from yeargroup 1 (*n* = 22), SS fry from yeargroup 1 (n = 22), RR fry from yeargroup 2 (*n* = 24), and SS fry from yeargroup 2 (n = 24). Genomic DNA from samples of fry fin tissue taken from individual fry within each group was then pooled at equimolar concentrations, resulting in four pools of genomic DNA. Each of these pools was then sequenced by Edinburgh Genomics (Edinburgh, UK) with 2 × 125 bp paired-end reads using HiSeq V4 chemistry, aiming for a mean coverage of each pool of 25×*.* The resulting sequencing reads of the four pools were trimmed from sequencing adapters, then aligned to the Atlantic salmon reference genome (Genbank accession GCA_000233375.4) using bwa-mem (PMID: 20080505). Resulting alignments in bam-format were subjected to duplicate removal using Picard (http://broadinstitute.github.io/picard/) and then variant calling using GATK [37] with the Unified Genotyper setting. GATK best practices were used for filtration of variants. Allelic depths observed for each pool at each SNP-position were exported from the vcf-file and were used in analysis to contrast the RR and SS pools within year group by means of determining their absolute differences in allele frequencies.

### 5.2 Disease challenge and gene expression analyses

To identify genes which appear to be differentially regulated between IPN resistant and susceptible individuals upon exposure to the virus, challenge experiments for analysis of gene expression patterns were set up as follows. 20 families of Atlantic salmon fry were challenged with IPNV (challenge method described in Houston et al [17]), with two replicate tanks of fry challenged for each family. It should be noted that these families were from yeargroup 2 as described above, but the families used for fine mapping and gene expression did not overlap. For each family, the level of mortality was averaged across the two replicate tanks, and mortalities across these families ranged from 0 to 34% upon challenge termination. Based on the levels of mortality, families J and N were designated susceptible, families Q and T appeared resistant and families I, P, B, O, D, S, C and L were designated as intermediate. To ascertain the QTL genotype of parents of challenged offspring within these families, a fin sample from each parent was removed and genotyped at the IPN QTL-linked microsatellite markers given in Houston et al. [17]. Families B and C were identified as ‘double heterozygote’ families where both parents were putative heterozygotes for the QTL, and, therefore, subsequent gene expression data was considered for these two families only.

### 5.3 IPNV testing

Fry mortalities and survivors from the challenged tanks and control tanks were tested for the presence of IPNV. Fry were weighed, homogenised using sterile pestle, mortar and sand then diluted 1:10 in cell culture medium. The homogenate was centrifuged at 2500 ×*g* for 15 min. at 4 °C then the supernatant removed and filtered through 0.45 μm filter (Whatman) before inoculation onto 24 h old confluent monolayers of CHSE-214 cells in 96-well cell culture trays for titration. Culture trays were incubated at 15 °C and titres read after 7 days. Wells showing positive cytopathic effect (CPE) for each sample were further tested by ELISA (Test-Line) to confirm the presence of IPNV. Subsequently, for the determination of viral load in the samples used for the microarray experiment, an RT-QPCR assay applied in an accredited commercial laboratory (Integrin Advanced Biosystems, UK) was used.

### 5.4 Microarray platform, hybridization, and quality filtering

RNA was extracted, purified, amplified and labelled as described in [20]. The microarray platform and methods for microarray hybridisation are described in [20]. Gene expression patterns between resistant and susceptible offspring within families B and C was analysed as follows. Each family was represented by three tanks each containing 100 fry, one of which was terminated and sampled at 1 day post-challenge (‘time point 1’), one at 7 days post-challenge (‘time point 2’) and one at 20 days post-challenge (‘time point 3’). In addition, a sample of 100 fry from all families was taken prior to challenge (‘time point 0’). To ascertain QTL genotype of sampled individuals at each time point, a fin sample from each offspring was removed and genotyped at the IPN QTL-linked microsatellite markers given in Houston et al. [17]. At each time point, RNA was extracted from six fish of each QTL genotype (i.e. homozygote resistant at the IPN QTL: RR; or homozygote susceptible at the IPN QTL: SS) and hybridised to the Agilent 44 K (Atlantic salmon) Oligo Array [38]. This microarray is comprised of 43,661 probes (partial gene sequences), representing ~90% of the known Atlantic salmon expressed sequence tags (ESTs) [39].

Significant differential expression of probes was determined by comparing the mean microarray signal across both time points, using a 3-way ANOVA [factors = QTL genotype (resistant vs. susceptible), family (B or C), and time point (0 or 1)]. To avoid exclusion of genes of potential biological relevance, a nominal threshold of *P* < 0.05 for significance was chosen (i.e. *P*-values were not corrected for multiple testing).

### 5.5 Virus and cell culture

Salmon head kidney, SHK-1 cells (ATCC 97111106) were propagated at 17.5 °C in L15 media supplemented with 5% FBS, 40 μM β-mercaptoethanol, 4 mM glutamine, and Pen Strep antibiotics. Cells were passaged using 0.25% trypsin/EDTA at 80% confluence, pelleted, and split 1:3. Fresh media was added in a 2:1 ratio with conditioned media. Chinook salmon embryo, CHSE-214 cells (ATCC 91041114) were propagated at 17.5 °C in L15 media supplemented with 10% FBS, 4 mM glutamine, and Pen Strep. Cells were passaged using 0.25% trypsin/EDTA at 80% confluence and split 1:6 in fresh media. IPNV VR1318 was provided by Marine Scotland as a crude isolate. Working stocks were established by infecting 80% confluent CHSE-214 cells at a very low MOI in normal cell culture conditions with 2% serum. At approximately 7 dpi, or when >50% of cells exhibited cytopathic effect, supernatant was harvested, debris was pelleted, and the viral stock was aliquoted and frozen at −80 °C. Viral stocks were titrated using plaque assay on CHSE-214. Infections with IPNV were performed on 80% confluent SHK-1 or CHSE-214 cells. Cells were seeded, incubated overnight, washed with PBS prior to overlay with virus diluted in serum free L15. After 2 h at 15 °C, viral inoculum was removed, washed with PBS and the cells were overlaid with 2% FBS media at 15 °C.

### 5.6 *Impact of nae1 and cdh1 knockout* in vitro

CRISPR-Cas9 gRNAs were designed for *nae1* and *cdh1* and selected for maximum on-target efficiency, and minimum off-targets, using the benchling (www.benchling.com) and the Synthego CRISPR design tools. *Nae1* KO and *cdh1* KO SHK-1 cells were produced by using method described in [26]. Briefly, SHK-1 cells were transfected with 1 μM Cas9 ribonucleoprotein targeting exon 2 of *nae1* or *cdh1* (Supplementary Table 1) by electroporation with 2 pulses at 1400 V for 20 ms. Genomic DNA was extracted at 7 days post electroporation, the target region was amplified by PCR (Supplementary Table 1), and gene-editing efficiency was assessed by Sanger sequencing and ICE analysis (https://ice.synthego.com), showing 94 and 93% editing efficiency in *nae1* KO and *cdh1* KO SHK-1 cells, respectively. The editing efficiency of both targets was stable up to 60 days post electroporation.

Between 30 and 40 days post electroporation KO SHK-1 cells as well as wild type SHK-1 cells were seeded in 48 well plates and incubated overnight. IPNV was inoculated at MOI of 0.01 in serum free L15 with Pen Strep for 2 h at 15 °C. Then, the viral inoculum was removed and cell monolayers were washed with PBS. 200 μL of L15 with 2% FBS, 40 μM β-mercaptoethanol and Pen Strep was added to each well and incubated at 15 °C. At 96 and 120 hpi, supernatants were collected and stored at −70 °C for TCID50 assays. Total RNAs from the cells were extracted using Direct-zol RNA microprep (Zymo Research, Irvine, USA) with DNase I treatment and stored at −70 °C for quantitative real-time PCR (qRT-PCR). Samples were collected from two and three independent challenge experiments with two and four technical replicates for 96 and 120 hpi, respectively.

To evaluate the viral load in cells, relative transcript level of IPNV VP2 to *ef1a* in the total RNAs was analysed by qRT-PCR using Luna Universal One-Step RT-qPCR reagent (NEB, Ipswich, USA) and LightCycler 480 Instrument (Roche, Basel, Switzerland) in duplicates. Each reaction consisted of 0.5 μL RNAs, 1× Reaction Mix, 1× Enzyme Mix, 0.4 μM each primer (Supplementary Table 2) and nuclease-free water up to 10 μL. The thermocycling initiated with reverse transcription at 55 °C for 10 min and initial denaturation at 95 °C for 1 min, followed by 40 cycles of denaturation at 95 °C for 10 s and extension at 60 °C for 30 s with plate read, and melt curve analysis. Efficiency and linearity (R^2^) of each primer pair were checked using serial dilution of total RNAs in duplicates. The relative viral transcript level of IPNV VP2 versus *ef1a* in the KO SHK-1 cells compared to wild type SHK-1 cells at each timepoint was calculated using 2^−ΔΔCT^. A Student *t-*test was used to assess the difference between the mean viral load values of KO versus WT cells. In a subsequent experiment, the impact of electroporation only (without editing) was assessed as another control and found to have no significant impact on viral load as assessed by RT-qPCR (data not shown).

The infectivity of viral output in the supernatants at 120 hpi was assessed by TCID50 on naïve CHSE-214 cells in 96 well plate format with 4 wells per dilution in 2% serum media. TCID50 was calculated by Reed and Muench method [40].

To assess the role of cdh1 in IPNV infection, antibody neutralisation was performed using serial 1:1 dilutions of BSA, IPNV-VP2 antibody and cdh1-specific antibody known to recognise Atlantic salmon Cdh1 [7] in a 96 well plate. SHK-1 cells were overlayed with media containing the serially dilute antibody or BSA and incubated at 15 °C for 2 h and were subsequently infected with IPNV at an MOI of 0.01. At 120 hpi, RNA was harvested from cells and IPNV viral load was assessed by qRT-PCR.

### 5.7 *Impact of inhibitor of Nae1 activity (MLN4924)* in vitro

Lyophilised MLN4924 was resuspended in DMSO. MLN4924 was titrated for cytotoxicity on CHSE-214 and SHK-1 cells. SHK-1 or CHSE-214 cells were seeded at 80% confluency and treated with 0 (DMSO only), 100 nM, 1 μM or 5 μM MLN4924 for 24 h prior to inoculation with IPNV at an MOI of 0.01. The impact of the MLN4924 on cell viability was assessed by sampling at 24, 48, 72, and 96 hpi and comparing cell survival in all challenged groups (including the DMSO control) versus the unchallenged control at the same timepoint.

To evaluate the infectivity of viral output, cells and supernatant were harvested at 120 hpi and assessed by TCID50 on naïve CHSE-214 cells. For semi-quantification of viral protein output, western blot against viral proteins was performed. At 120 hpi, supernatant from a 150 mm dishes containing SHK-1 cells treated with either 100 nM MLN4924 or DMSO for 24 h before infection with IPNV at an MOI of 0.01 was collected, sterile filtered, and ultra-centrifuged at 22000 x*g* for 1 h. The ultra-centrifuged virus pellet from the supernatant was resuspended in Laemmli buffer. The cell-associated virus was also analysed by harvesting cells in Laemmli buffer. These samples were separated by PAGE (4–15% Mini-Protean, BIORAD), transferred onto nitrocellulose membrane, and the viral protein was visualised using a monoclonal antibody that recognises all IPN viral proteins, and secondary LICOR antibodies.

## Data access

Raw sequence data from this article are available at NCBI Short Read Archive under project number PRJNA614520, with other raw data provided as supplementary material.

## Author contributions

Conceptualization: JP, JWJ, RLG, JBT, AH, DWJ, RKP, SCB, JEB, RDH

Formal analysis: JP, YHJ, RLG, CR, JEB, DR

Project administration: RH, JEB

Writing - original draft: JP, YHJ, DR, RDH

Writing - review & editing: All authors

## Competing interests statement

The authors do not have any competing interests.

## Ethics statement

All challenge experiments were performed under approval of Cefas Ethical Review Committee and complied with the Animals Scientific Procedures Act. Fish were euthanised using a non-schedule 1 method under a procedure specifically listed on the appropriate Home Office (UK) license.

## Appendix A Supplementary data

Supplementary File 1 KASP assay genotypes for 21 SNPs in the IPNV QTL region for homozygous resistant (RR) and homozygous susceptible (SS) animals. Image 1

Supplementary File 2 Differential expression between resistant and susceptible fish. The microarray probe, gene annotation, log2 fold change, p-value and genomic position are shown for those genes showing significant expression differences between resistant and susceptible samples at control, 1 dpi and 7 dpi timepoints. Image 2

Supplementary Fig. 1 IPNV viral load at day 1, day 7, and day 21 post infection by genotype (RR, RS, and SS). Image 3

Supplementary Fig. 2 IPNV viral load after chemical inhibition of Nae1 using MLN4924 in SHK-1 and CHSE-214. Image 3

Supplementary Fig. 3 Cell viability in SHK-1 and CHSE-214 after MLN4924 treatment and IPNV challenge. Image 3

Supplementary Fig 4 Antibody neutralisation test of Cdh1. SHK-1 cells and corresponding inocula were treated with a dilution series of antibodies against BSA, IPNV VP2, or Cdh1. Cells were infected at an MOI of 0.01, and at 120 hpi IPNV viral load was ascertained by qRT-PCR. Image 3

Supplementary File 3 Samples used for the microarray experiment. Sample ID, Family ID, QTL genotype, IPNV infection timepoint, tank, and IPNV resistance phenotype are shown for each sample. Image 4

Supplementary File 4 Microarray expression data. Cy3 fluorescence (“rProcessedSignal(raw)”), Cy5 fluoresence (“gProcessedSignal(raw)”) and normalized expression (“cy3ByCy5Signal(normalized)”) are shown for every sample in supplementary file Y and every probe in the microarray. The annotation of every probe is also shown. Image 5

Supplementary Table 1 crRNAs and primers used for nae1 and cdh1 knockout, respectively. Supplementary Table 1

Supplementary Table 2 Primers used for qRT-PCR for assessing IPNV viral load. Supplementary Table 2
