# Supplementary figures and images for: The nedd-8 activating enzyme gene underlies genetic resistance to infectious pancreatic necrosis virus in Atlantic salmon
Source: Genomics. 2021 Nov;113(6):3842–50. doi: 10.1016/j.ygeno.2021.09.012 (PMC8682971; doi:10.1016/j.ygeno.2021.09.012)

## Slide 1
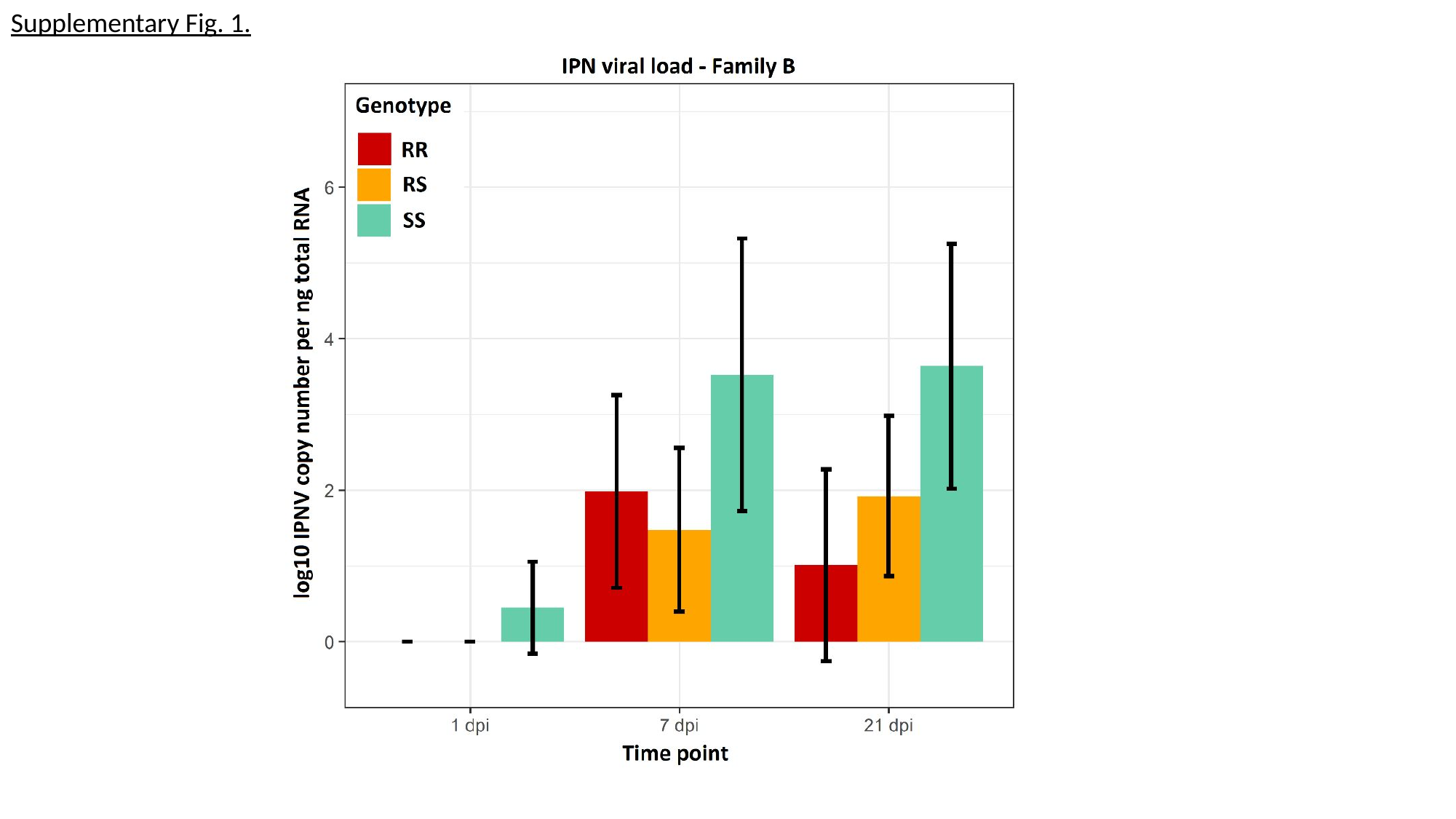

Supplementary Fig. 1.

Supplement: Supplementary Fig. 1 — IPNV viral load at day 1, day 7, and day 21 post infection by genotype (RR, RS, and SS). [file mmc3.pptx]

## Slide 1
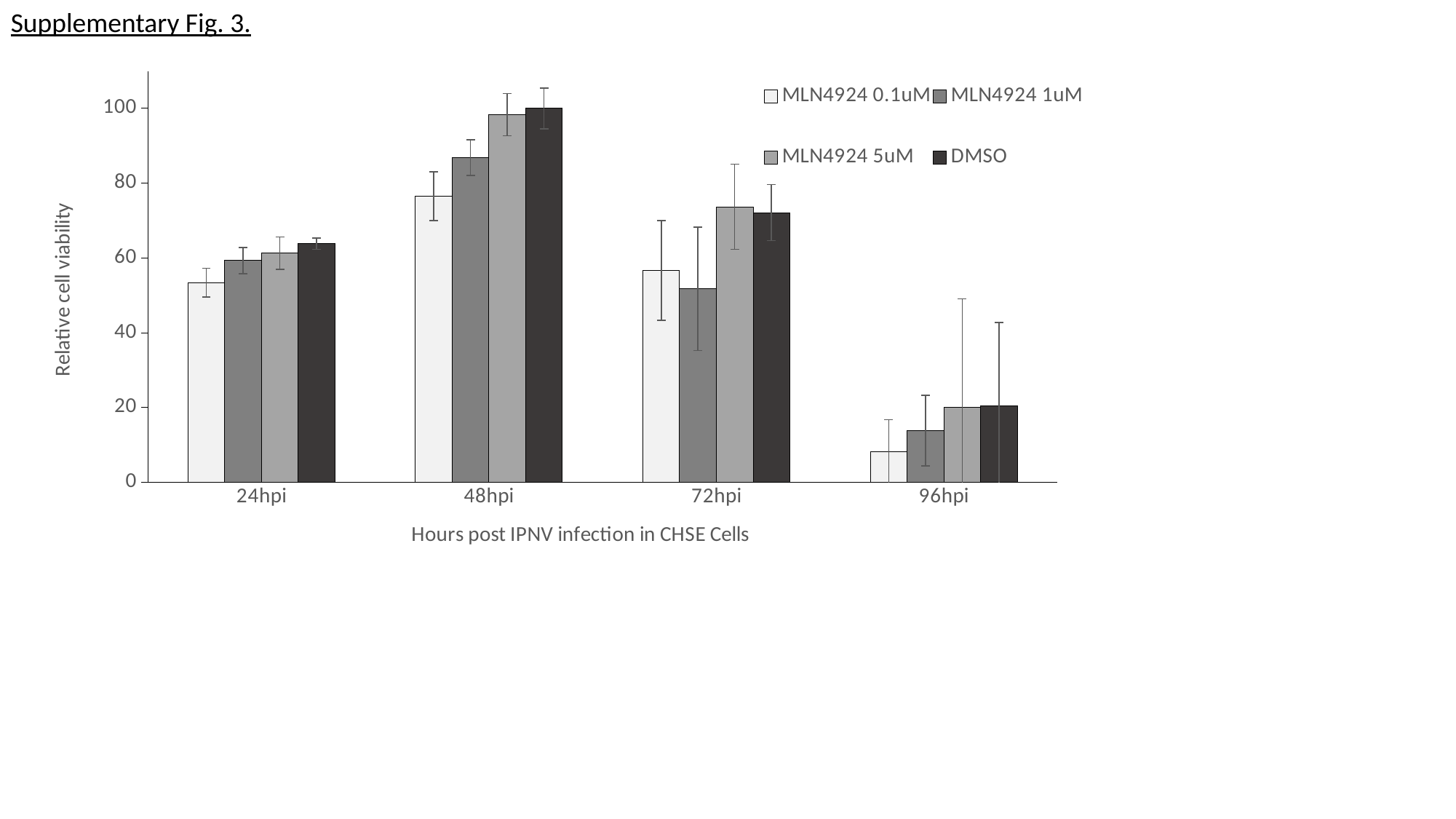

Supplementary Fig. 3.
### Chart
| Category | MLN4924 | MLN4924 | MLN4924 | DMSO |
|---|---|---|---|---|
| 24hpi | 53.41372423155979 | 59.32819213297936 | 61.277278145947164 | 63.79490815397657 |
| 48hpi | 76.55471732717224 | 86.81970982337562 | 98.35777565549186 | 100.0 |
| 72hpi | 56.650085997311606 | 51.770046096348295 | 73.69175611207162 | 72.1430216927939 |
| 96hpi | 8.100521799032446 | 13.826667889341927 | 20.11643745233244 | 20.539043226789115 |

Supplement: Supplementary Fig. 3 — Cell viability in SHK-1 and CHSE-214 after MLN4924 treatment and IPNV challenge. [file mmc5.pptx]

## Slide 1
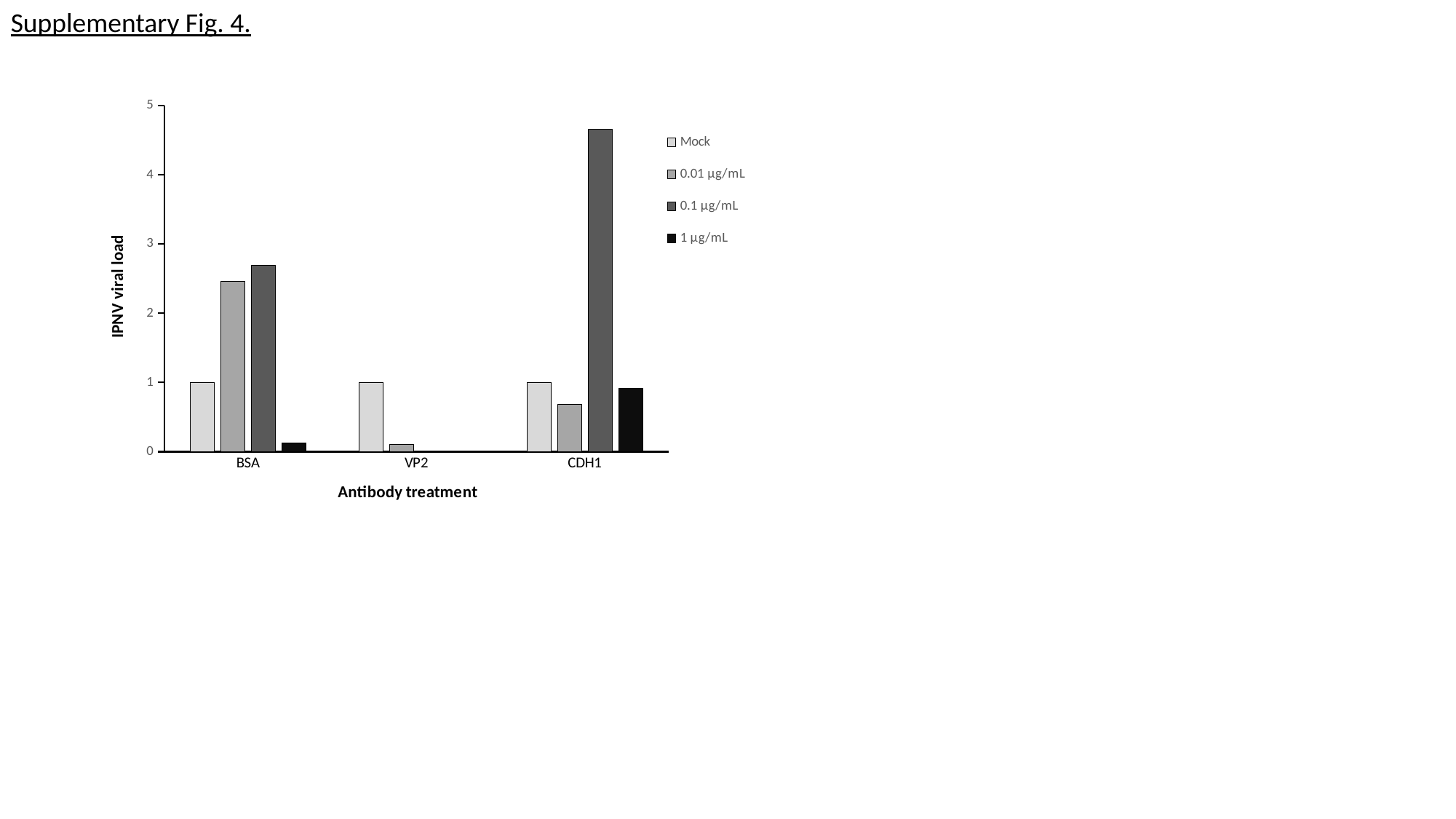

Supplementary Fig. 4.
### Chart
| Category | Mock | 0.01 µg/mL | 0.1 µg/mL | 1 µg/mL |
|---|---|---|---|---|
| BSA | 1.0 | 2.462288827 | 2.694467154 | 0.126744935 |
| VP2 | 1.0 | 0.10153155 | 0.000465155 | 0.003670011 |
| CDH1 | 1.0 | 0.683020128 | 4.658934346 | 0.91383145 |

Supplement: Supplementary Fig 4 — Antibody neutralisation test of Cdh1. SHK-1 cells and corresponding inocula were treated with a dilution series of antibodies against BSA, IPNV VP2, or Cdh1. Cells were infected at an MOI of 0.01, and at 120 hpi IPNV viral load was ascertained by qRT-PCR. [file mmc6.pptx]
