# Supplementary material for: The nedd-8 activating enzyme gene underlies genetic resistance to infectious pancreatic necrosis virus in Atlantic salmon
Source: Genomics. 2021 Nov;113(6):3842–50. doi: 10.1016/j.ygeno.2021.09.012 (PMC8682971; doi:10.1016/j.ygeno.2021.09.012)
Supplement: Supplementary Fig. 2 — IPNV viral load after chemical inhibition of Nae1 using MLN4924 in SHK-1 and CHSE-214. [file mmc4.pptx]

## Slide 1
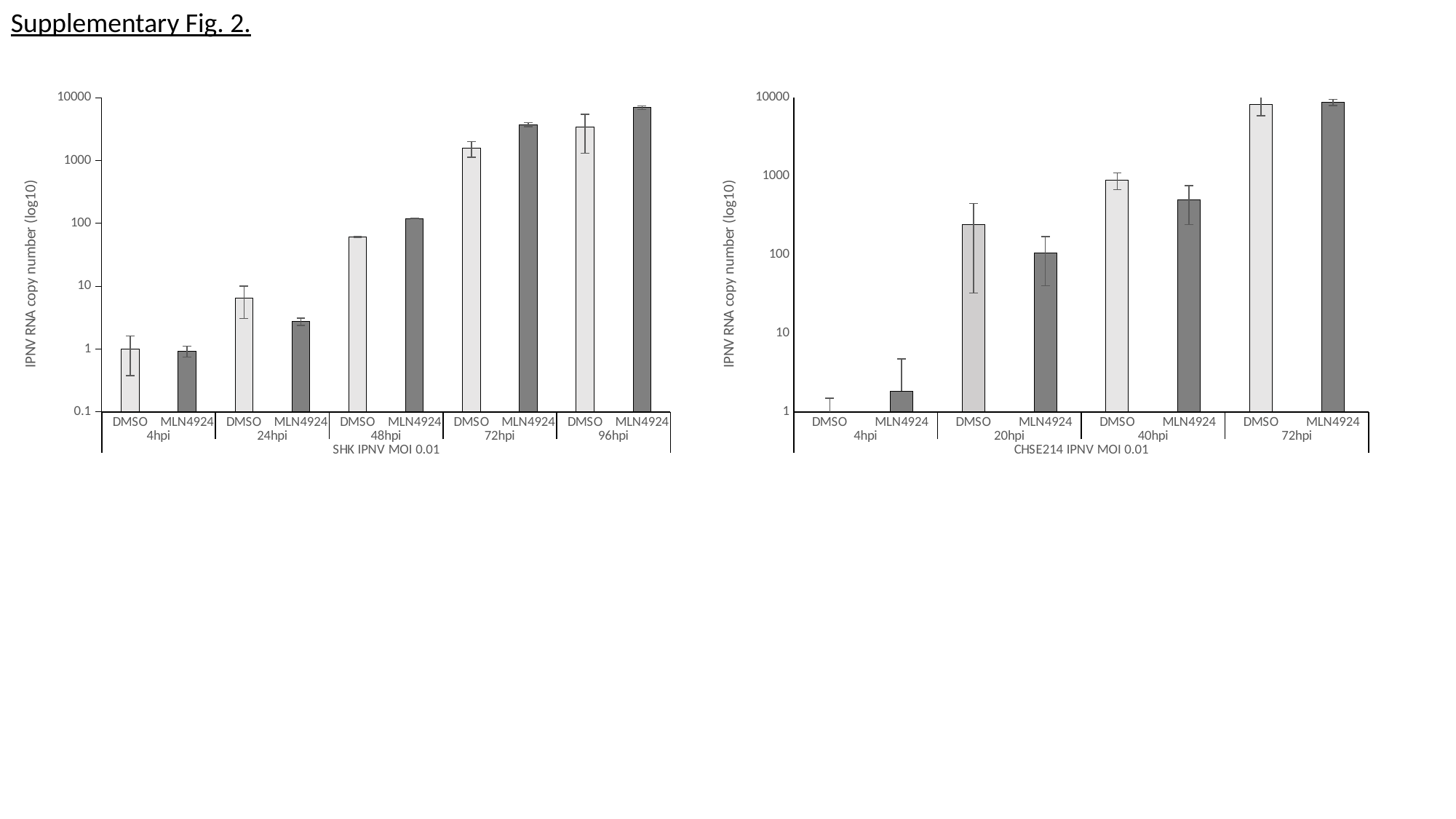

Supplementary Fig. 2.
### Chart
| Category | |
|---|---|
| DMSO | 1.0 |
| MLN4924 | 0.929804942613163 |
| DMSO | 6.520578659221014 |
| MLN4924 | 2.7510836362794846 |
| DMSO | 60.33821029876739 |
| MLN4924 | 119.84284767161506 |
| DMSO | 1568.3153890246476 |
| MLN4924 | 3730.1036383927426 |
| DMSO | 3373.4288067279786 |
| MLN4924 | 6936.537598873813 |
### Chart
| Category | |
|---|---|
| DMSO | 1.0 |
| MLN4924 | 1.8340080864093453 |
| DMSO | 241.35284119506923 |
| MLN4924 | 105.41965021024919 |
| DMSO | 888.3596156437177 |
| MLN4924 | 503.2041464551692 |
| DMSO | 8248.97986606463 |
| MLN4924 | 8719.320534657916 |
