# Supplementary material for: The nedd-8 activating enzyme gene underlies genetic resistance to infectious pancreatic necrosis virus in Atlantic salmon
Source: Genomics. 2021 Nov;113(6):3842–50. doi: 10.1016/j.ygeno.2021.09.012 (PMC8682971; doi:10.1016/j.ygeno.2021.09.012)
Supplement: Supplementary Table 1 — crRNAs and primers used for nae1 and cdh1 knockout, respectively. [file mmc9.docx]

Supplementary Table 1. crRNAs and primers used for *nae1* and *cdh1* knockout, respectively

|  | crRNA | Gene ID | Fw (5’-3’) | Rv (5’-3’) | Amplicon (bp) | Annealing temp (°C) |
| --- | --- | --- | --- | --- | --- | --- |
| *nae1* | TGATCAATTCCACAGCATCT | 100194854 | ATGCCAGCCAACCAGCATGCTT | TTCCGACGTCTTCCCCGGAGAC | 580 | 69 |
| *cdh1* | TCGGAGTCAACATGTCTACC | 106587268 | CCACATTTCGCAATCGGGTGAA | ATTTCCCAGTCGGAGCTCGTTT | 416 | 56 |
