# Supplementary material for: The nedd-8 activating enzyme gene underlies genetic resistance to infectious pancreatic necrosis virus in Atlantic salmon
Source: Genomics. 2021 Nov;113(6):3842–50. doi: 10.1016/j.ygeno.2021.09.012 (PMC8682971; doi:10.1016/j.ygeno.2021.09.012)
Supplement: Supplementary Table 2 — Primers used for qRT-PCR for assessing IPNV viral load. [file mmc10.docx]

Supplementary Table 4. Primers used for qRT-PCR for assessing IPNV viral load

|  | Fw (5’-3’) | Rv (5’-3’) | Amplicon (bp) | Efficiency | R^2^ | Gene ID/ref |
| --- | --- | --- | --- | --- | --- | --- |
| *ef1a* | GGCTGGTTCAAGGGATGGA | CAGAGTCACACCATTGGCG | 60 | 1.98 | 0.999 | AF321836 |
| IPNV VP2 | GACCAAGTTCGACTTCCAGC | ATCGGCTTGGTGATGTTCTC | 156 | 1.96 | 0.9997 | FN257531.1 ^22^ |
